# Peripheral organ crosstalk in the regulation of ovarian endocrine function and reproductive homeostasis

**DOI:** 10.1093/lifemedi/lnaf029

**Published:** 2025-08-28

**Authors:** Lixuan Huang, Lei Xu, Chuyu Yun, Jie Qiao, Xinyu Qi

**Affiliations:** State Key Laboratory of Female Fertility Promotion, Center for Reproductive Medicine, Department of Obstetrics and Gynecology, Peking University Third Hospital, Beijing 100191, China; National Clinical Research Center for Obstetrics and Gynecology (Peking University Third Hospital), Beijing 100191, China; Key Laboratory of Assisted Reproduction (Peking University), Ministry of Education, Beijing 100191, China; Department of Pharmacy, Peking University Third Hospital, Beijing 100191, China; State Key Laboratory of Female Fertility Promotion, Center for Reproductive Medicine, Department of Obstetrics and Gynecology, Peking University Third Hospital, Beijing 100191, China; National Clinical Research Center for Obstetrics and Gynecology (Peking University Third Hospital), Beijing 100191, China; Key Laboratory of Assisted Reproduction (Peking University), Ministry of Education, Beijing 100191, China; Beijing Key Laboratory of Reproductive Endocrinology and Assisted Reproductive Technology, Beijing 100191, China; State Key Laboratory of Female Fertility Promotion, Center for Reproductive Medicine, Department of Obstetrics and Gynecology, Peking University Third Hospital, Beijing 100191, China; National Clinical Research Center for Obstetrics and Gynecology (Peking University Third Hospital), Beijing 100191, China; Key Laboratory of Assisted Reproduction (Peking University), Ministry of Education, Beijing 100191, China; Beijing Key Laboratory of Reproductive Endocrinology and Assisted Reproductive Technology, Beijing 100191, China; State Key Laboratory of Female Fertility Promotion, Center for Reproductive Medicine, Department of Obstetrics and Gynecology, Peking University Third Hospital, Beijing 100191, China; National Clinical Research Center for Obstetrics and Gynecology (Peking University Third Hospital), Beijing 100191, China; Key Laboratory of Assisted Reproduction (Peking University), Ministry of Education, Beijing 100191, China; Beijing Key Laboratory of Reproductive Endocrinology and Assisted Reproductive Technology, Beijing 100191, China

**Keywords:** ovarian dysfunction, GnRH pulse frequency, gut microbiota dysbiosis, chronic inflammation, steroid hormone metabolism

## Abstract

Ovarian function is increasingly recognized as a systemic process influenced by cross-organ communication, yet knowledge regarding the underlying mechanisms remains fragmented. In addition to the ovary, multiple organs, including the hypothalamus, adipose tissue, gut, liver, thyroid, adrenal gland, and heart, converge to regulate ovarian function. These interactions span endocrine signals (e.g. insulin hormones), immune mediators (e.g. cytokines), and metabolic cues (e.g. adipokines), collectively impacting folliculogenesis, steroidogenesis, and ovulation. Importantly, these interactions suggest novel therapeutic targets to mitigate ovarian dysfunction. However, causal relationships and organ-specific contributions require further mechanistic exploration. This synthesis underscores the necessity of a multisystem perspective in both the research and clinical management of ovarian disorders.

## Introduction

The ovaries, as core organs of the female reproductive and endocrine systems, play a vital role in not only periodic ovulation but also in maintaining overall endocrine homeostasis and metabolic balance through the secretion of bioactive substances such as estrogen and progesterone. Ovarian reserve refers to the number and quality of follicles and plays a fundamental role in supporting normal ovarian function, including fertility and hormone production [1]. However, ovarian function is also influenced by neuroendocrine, immune, and metabolic systems. Recent research has broken traditional cognitive limitations, revealing that the ovary is not an isolated “autonomous organ” but operates through a complex, multi-dimensional inter-organ dialogue.

Among the various clinical phenotypes of ovarian dysfunction, polycystic ovary syndrome (PCOS) and ovarian aging represent two opposing extremes of follicular reserve status. PCOS, the most common reproductive endocrine disorder in women of reproductive age (with a prevalence of approximately 6%–20%), is characterized by hyperandrogenism, oligo-ovulation, and polycystic ovarian morphology. Approximately 50%–80% of patients also exhibit metabolic abnormalities such as insulin resistance [2]. In PCOS, the ovaries typically contain many small antral follicles (2–9 mm in diameter), reflecting preserved or even excessive follicular reserve. However, most of these follicles fail to develop into dominant follicles, resulting in chronic anovulation. In contrast, ovarian aging is a physiological process in which the natural depletion of ovarian reserve marks the inevitable endpoint of a woman’s reproductive lifespan. Notably, the ovary is the first organ to undergo aging in women, with ovarian function typically beginning to decline around the age of 35, highlighting the importance of implementing strategies to delay ovarian aging [3]. However, factors such as environmental pollutants, metabolic disorders, and iatrogenic damage have contributed to the rising incidence of premature ovarian insufficiency (POI), with 3.7% of women experiencing ovarian function decline before the age of 40 [4]. These contrasting states offer a valuable framework for examining how peripheral organ signals regulate ovarian function under different follicular reserve conditions.

## Overview

**Figure 1.**
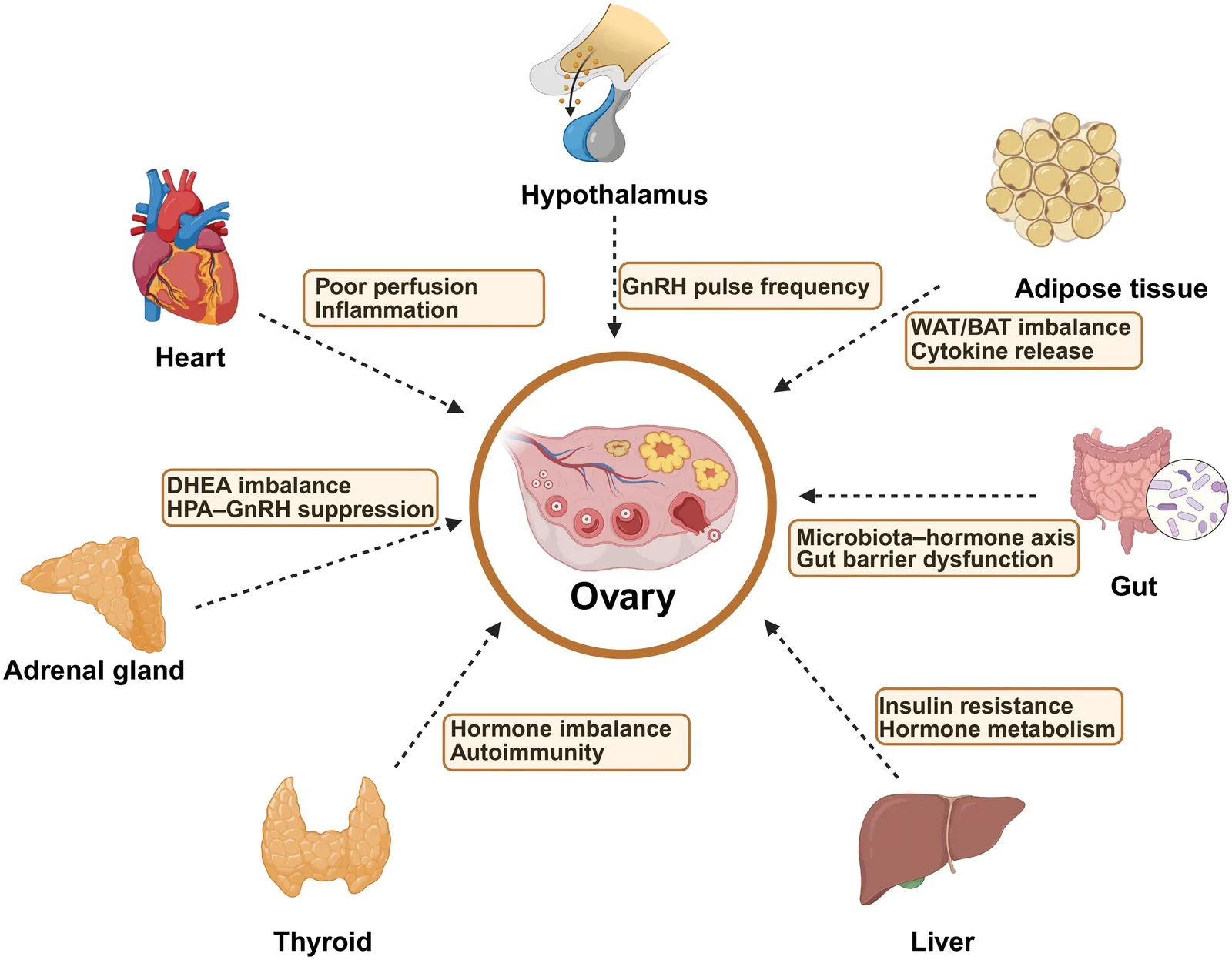
The interactions between ovarian dysfunction and multiple organ systems. Ovarian function is dynamically regulated by peripheral organs, including the hypothalamus, adipose tissue, gut, liver, thyroid, adrenal glands, and heart. These organs influence ovarian physiology through endocrine, immune, and metabolic pathways. Key mechanisms include hypothalamic regulation of GnRH pulsatility; WAT/BAT imbalance and cytokine release; gut barrier dysfunction and microbiota-hormone interactions; hepatic insulin resistance and altered hormone metabolism; thyroid hormonal imbalance and autoimmunity; suppression of the HPA axis mediated by adrenal DHEA; and cardiac hypoperfusion and inflammation. Disruptions in these inter-organ signals may impair ovarian function, manifesting as ovulatory dysfunction and hormonal dysregulation.

The ovary plays a central role not only in reproduction but also as a critical endocrine organ regulating systemic metabolism, immune function, and hormonal homeostasis. In recent years, accumulating evidence has demonstrated that ovarian function is dynamically regulated through interactions with multiple peripheral organs, involving hormonal signaling, metabolic crosstalk, and immune pathways (Fig. 1).

## Hypothalamus–ovary crosstalk: GnRH pulsatility dysregulation from PCOS to reproductive senescence

**Figure 2.**
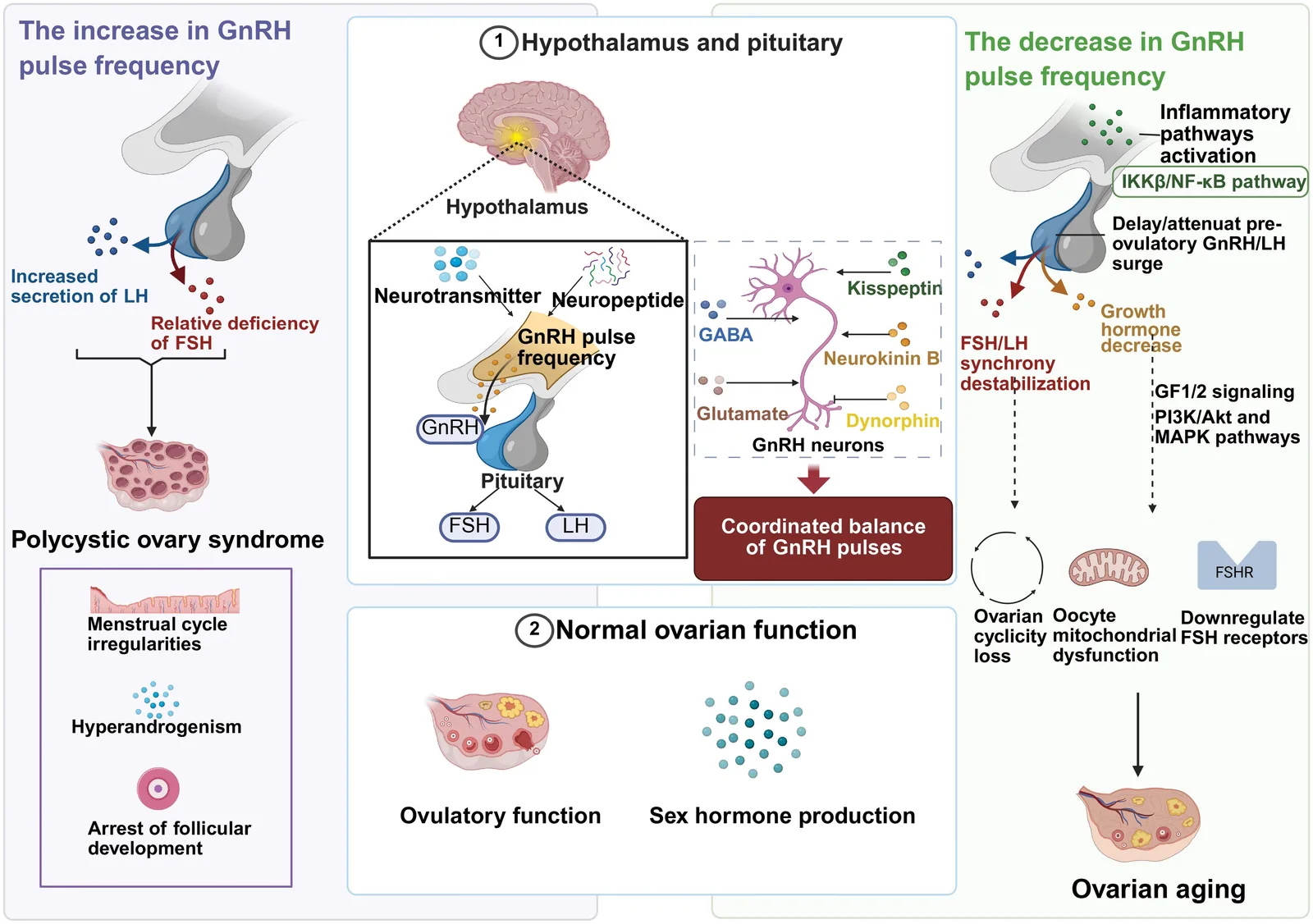
Mechanisms by which alterations in GnRH pulse frequency affect ovarian function. Under physiological conditions, the hypothalamic–pituitary axis regulates the pulsatile release of GnRH through the coordinated actions of neurotransmitters and neuropeptides, such as γ-aminobutyric acid (GABA), glutamate, kisspeptin, neurokinin B, and dynorphin. This coordination maintains a balanced secretion of FSH and LH, thereby supporting normal ovarian function, including ovulatory function and sex hormone production. An abnormal increase in GnRH pulse frequency leads to elevated LH secretion and a relative deficiency of FSH, contributing to the development of PCOS, which is characterized by menstrual irregularities, hyperandrogenism, and arrested follicular development. Conversely, a decrease in GnRH pulse frequency activates inflammatory pathways (e.g. IKKβ/NF-κB) and reduces growth hormone levels, resulting in a delayed or attenuated pre-ovulatory GnRH/LH surge, disruption of FSH/LH synchrony, loss of ovarian cyclicity, oocyte mitochondrial dysfunction, and downregulation of FSH receptors, ultimately accelerating ovarian aging.

The hypothalamic regulation of pulsatile gonadotropin-releasing hormone (GnRH) secretion involves complex interplay between neurotransmitters (e.g. kisspeptin) and neuropeptides (e.g. neurokinin B), which govern critical reproductive processes, including puberty onset and cyclic ovulation [5]. Importantly, the GnRH pulse frequency is decoded by pituitary gonadotropes through differential activation of intracellular signaling cascades, ultimately affecting the luteinizing hormone (LH)/follicle-stimulating hormone (FSH) secretion ratio [6]. Clinical observations have revealed that accelerated GnRH pulse frequencies in patients with conditions such as PCOS drive aberrant LH hypersecretion and subsequent ovarian hyperandrogenism [7], whereas diminished pulsatility characterizes hypoestrogenic states such as premature ovarian insufficiency [8]. These findings highlight the central role of GnRH pulse pattern modulation in maintaining ovarian homeostasis, warranting further exploration of its diagnostic and therapeutic potential (Fig. 2).

PCOS, a globally prevalent endocrine-metabolic disorder affecting 6%‒20% of reproductive-aged women [9], is characterized by Rotterdam diagnostic criteria including hyperandrogenism (clinical or biochemical), oligo-anovulation, and polycystic ovarian morphology on ultrasonography. Notably, emerging evidence suggests that hypothalamic dysfunction plays a central role in PCOS pathogenesis, where accelerated GnRH neuronal pulsatility (approximately 1 pulse/60 min) drives excessive LH secretion from pituitary gonadotropes [6]. This neuroendocrine dysregulation creates a self-perpetuating cycle: elevated LH levels stimulate ovarian theca cell androgen overproduction, whereas relative FSH deficiency impairs follicular maturation, ultimately manifesting as the hallmark polycystic ovarian morphology [10]. Recent studies have shown that targeting excessive activation of GnRH neurons can significantly increase LH secretion, thereby promoting the development of a PCOS-like phenotype in mice comprising prolonged hyperandrogenism, menstrual cycle disruption, and ovarian follicular stagnation [6]. These findings suggest that the overactivation of GnRH neurons contributes to the onset of PCOS-like ovarian dysfunction. Elevated GnRH pulsatility in PCOS patients significantly impacts ovarian and endocrine function, and this high-frequency pulsatility is not coincidental but is closely associated with abnormalities in various neuroendocrine regulatory factors. The following section analyzes the potential causes of elevated GnRH pulsatility.

The pulsatile secretion of GnRH is tightly regulated by hypothalamic neurocircuitry involving γ-aminobutyric acid (GABA), glutamate, and neuropeptide Y (NPY), the dysregulation of which may contribute to PCOS pathogenesis. The hypothalamic regulation of GnRH pulsatility in PCOS patients involves coordinated dysregulation of key neurotransmitter systems. Emerging evidence challenges classic neurotransmission paradigms, revealing that GABA exerts paradoxical excitatory effects on adult GnRH neurons, with PCOS models demonstrating 40% increased synaptic density between arcuate nucleus (ARC) GABAergic neurons and GnRH neurons, which correlates with elevated LH pulse frequency [11–14]. This GABAergic hyperactivity synergizes with glutamatergic dysregulation, in which prenatal androgen exposure induces persistent N-methyl-D-aspartate (NMDA) receptor overexpression and astrocyte-mediated glutamate clearance impairment, collectively driving GnRH neuronal hyperexcitability [12]. Crucially, disruption of glial-GnRH neuron interactions in the arcuate nucleus-median eminence (ARC-ME) region leads to pathological acceleration of the GnRH pulse frequency, directly contributing to PCOS phenotype progression [15]. Concurrently, NPY signaling disruption manifests as phase-advanced diurnal rhythms and selective Y2 receptor expression downregulation [16], which destabilizes kisspeptin-mediated GnRH pulse synchronization [17, 15].

The hypothalamic kisspeptin system governs GnRH pulse generation through anatomically and functionally distinct neuronal populations. Anteroventral periventricular nucleus (AVPV) kisspeptin-expressing neurons mediate positive feedback to estrogen signaling to trigger preovulatory GnRH/LH surges, whereas ARC KNDy neurons (co-expressing kisspeptin, neurokinin B, and dynorphin) maintain basal pulsatility via balanced neurokinin B (NK3R)-mediated synchronization and dynorphin (KOR)-dependent pulse termination [18].

PCOS arises from hypothalamic dysregulation, in which hyperactive GnRH pulsatility disrupts pituitary gonadotropin secretion. Intriguingly, hypothalamic control over ovarian function undergoes recalibration due to age: decreasing sensitivity of hypothalamic neurons to sex steroid feedback during perimenopause destabilizes GnRH pulse generator rhythmicity [19]. This neuroendocrine dissonance accelerates follicular reserve depletion and initiates reproductive senescence. Ultimately, the erosion of hypothalamic‒ovarian crosstalk propels the transition to menopause. Studies have shown that the decline in hypothalamic function in perimenopausal women is closely associated with ovarian aging. Specifically, aging disrupts GnRH pulsatility, notably delaying/attenuating the preovulatory GnRH/LH surge. Middle-aged rodents exhibit reduced GnRH neuron excitability and enhanced inhibitory signaling, ultimately diminishing LH surges and contributing to the loss of ovarian cyclicity [20]. Research has indicated that early changes in hypothalamic aging occur in animals with regular reproductive cycles, suggesting that the hypothalamic aging process begins before irregular cycles emerge [21].

Hypothalamic aging drives ovarian senescence through convergent inflammatory and metabolic perturbations. Chronic activation of the IKKβ/NF-κB pathway establishes a neuroinflammatory milieu [22]. Comparative studies of hypothalamic NF-κB activity in young and middle-aged mice have shown minimal activity in young mice, whereas middle-aged mice exhibit a significant increase in activity that intensifies with age [21]. This inflammatory cascade transcriptionally represses GnRH via direct promoter binding—reducing transcriptional activity by 50%—while impairing kisspeptin neuron function [21, 22]. Consequently, disrupted GnRH pulsatility destabilizes FSH/LH synchrony, triggering follicular apoptosis through the deprivation of trophic support.

Parallel to inflammatory dysregulation, age-related decay of the hypothalamic‒pituitary‒ovarian metabolic axis exacerbates reproductive decline. Growth hormone-releasing hormone (GHRH) neuron degeneration in the arcuate nucleus diminishes growth hormone (GH) secretion, which normally sustains follicular vitality via ovarian insulin-like growth factor (IGF)1/2 signaling [23]. IGF1/2 engagement of the PI3K/Akt and mitogen-activated protein kinase (MAPK) pathways improves oocyte mitochondrial function in GH-supplemented cohorts while upregulating FSH receptor expression to lower follicular atresia thresholds [24, 25]. The perimenopausal decline in circulating IGF1 and GH starves follicles of these survival signals, manifesting as impaired oocyte maturation [26]. Therapeutic GH administration (4 IU/day) partially restores this axis, resulting in a significantly greater percentage of high-quality blastocysts (34.27% vs. 23.90%) among women aged 35–40 years undergoing *in vitro* fertilization (IVF) [27], underscoring the hypothalamus as a pivotal coordinator of ovarian aging trajectory.

In addition to hormonal and neuropeptide regulation, autophagy exerts dual effects on ovarian aging through its critical role in maintaining cellular homeostasis. This self-degradative process has paradoxical impacts during folliculogenesis: physiological autophagy sustains follicular energy equilibrium, whereas hyperactivation triggers granulosa cell apoptosis and accelerates follicular atresia [24]. Notably, autophagic deficiency disrupts follicular maturation, which is characterized by depleted primordial/antral follicle reserves and elevated atretic follicle ratios. Mechanistically, endocrine regulators (notably insulin) orchestrate PI3K/Akt/mTOR signaling through IRS1 activation, with mTOR hyperactivation suppressing autophagic flux and propagating proteotoxic stress during ovarian aging [28].

Ovarian senescence is a hypothalamus-driven process in which central regulatory mechanisms orchestrate peripheral gonadal aging. Emerging evidence positions the hypothalamus as the master regulator through three interlinked pathways: neuroinflammation-triggered oxidative stress, endocrine hormone dysregulation, and autophagic impairment. Specifically, age-related hypothalamic inflammation disrupts GnRH pulsatility, whereas autophagic dysfunction impairs nutrient-sensing pathways, including the PI3K/Akt/mTOR pathway, synergistically exacerbating follicular depletion. The perimenopausal transition epitomizes this central‒peripheral crosstalk, as hypothalamic‒pituitary signaling decay precipitates GH/IGF axis collapse and ovarian reserve exhaustion. Crucially, ovarian aging reflects hierarchical failure of the hypothalamic command center rather than isolated gonadal dysfunction. Deciphering this neuroendocrine hierarchy reveals novel avenues for preserving ovarian longevity through hypothalamic rejuvenation strategies.

## Adipose tissue–ovary crosstalk: the invisible link between obesity and ovarian dysfunction

To date, the proportion of obesity in the population and the increasing trend in prevalence are becoming increasingly evident. The global obesity rate is rising rapidly, and obesity has already become an epidemic [29]. The escalating obesity pandemic poses unprecedented challenges to women’s reproductive health, with over 40% of women of childbearing age currently affected by excessive adiposity [30]. Projections indicate a concerning trajectory: the global population with an elevated body mass index (BMI ≥ 25 kg/m²) is anticipated to rise from 2.2 billion (42% of adults) in 2020 to 3.3 billion (54%) by 2035 [31]. This metabolic perturbation profoundly impacts ovarian function, correlating with a threefold increase in infertility rates [32], prolonged conception intervals [33], and compromised IVF outcomes, including reduced oocyte retrieval counts and diminished live birth probabilities (RR = 0.85, 95% CI: 0.82–0.87) [34, 35].

Adipose tissue depots exhibit remarkable functional diversity across anatomical locations. White adipose tissue (WAT), comprising more than 90% of adult fat mass, operates as both an energy reservoir and an endocrine organ through secreted mediators such as leptin (proinflammatory), adiponectin (insulin-sensitizing), and resistin (insulin resistance-associated) [36]. Additionally, protein tyrosine phosphatase 1B (PTP1B), a negative regulator of leptin and insulin signaling, has been shown to exacerbate systemic metabolic inflammation and may accelerate multi-organ aging, including that of the ovary [37]. In contrast, brown adipose tissue (BAT) predominantly regulates thermogenesis via mitochondrial uncoupling protein 1 (UCP1)-mediated nonshivering thermogenesis, with distinct anatomical distributions—human BAT is concentrated in the supraclavicular and paravertebral regions, whereas rodent BAT is located in the interscapular and perirenal areas [38–40]. Notably, the peri-ovarian adipose depot (POAT) has unique spatial and functional interactions with ovarian physiology.

Experimental models have elucidated adipose‒ovarian crosstalk through various pathways. Surgical POAT removal in murine models reduces ovarian mass by 20.7% (5.94 vs. 4.71 mg, *P* < 0.05) and disrupts folliculogenesis, resulting in decreased antral follicle numbers, increased atresia rates, and impaired oocyte competence [41]. In diet-induced obesity models (high-fat/high-sucrose feeding), ovarian function is disrupted, manifesting as irregular estrous cycles and impaired folliculogenesis [42]. Mechanistically, adipocyte-derived extracellular vesicles from obese individuals carry an elevated abundance of miR-26b, which suppresses granulosa cell proliferation by targeting JAG1/Notch signaling, thereby contributing to steroidogenic dysregulation [43].

**Figure 3.**
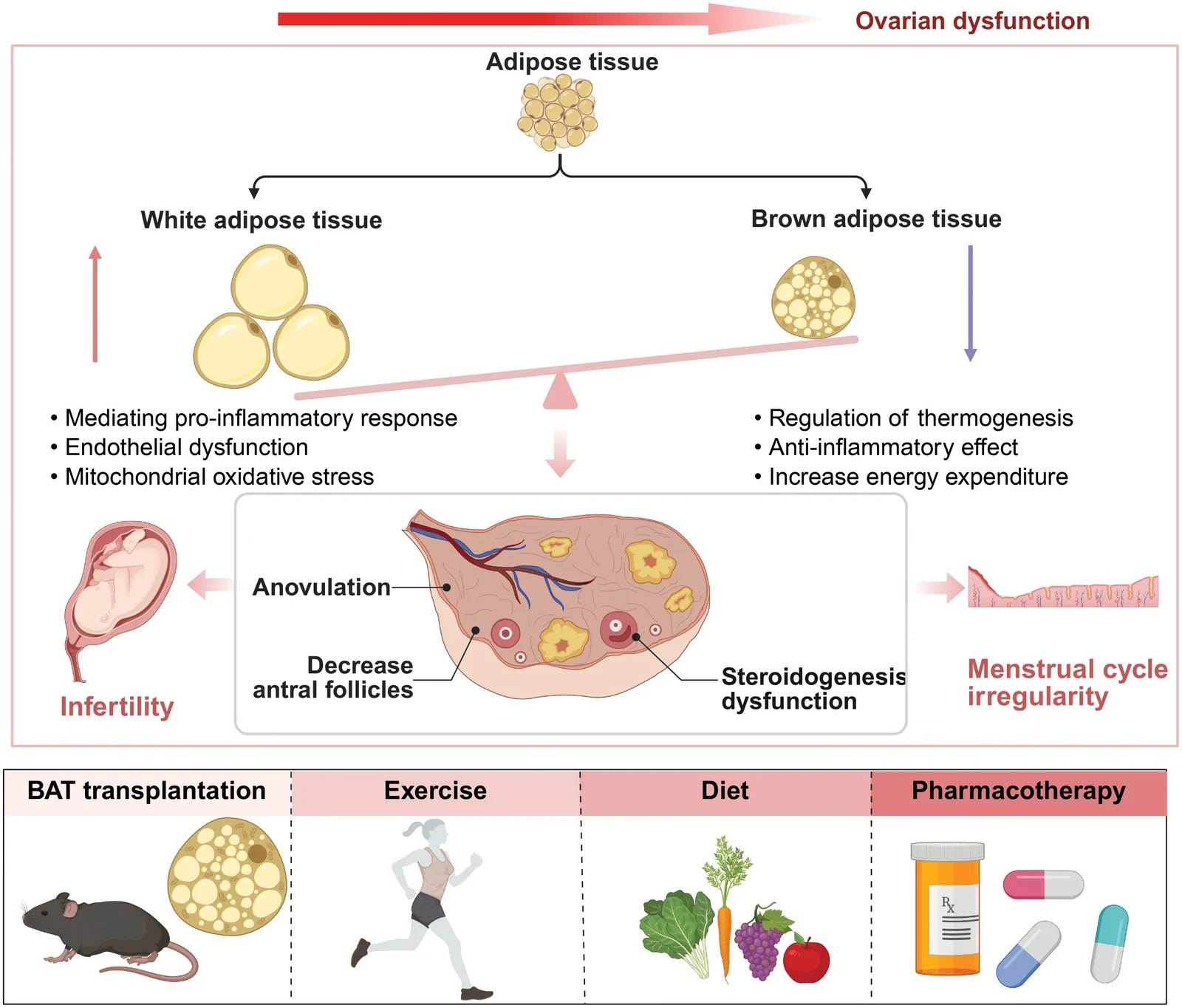
The role of adipose tissue dysregulation in ovarian dysfunction and potential intervention strategies. Adipose tissue regulates ovarian function through multiple mechanisms. Expansion of WAT promotes ovarian functional impairment by mediating pro-inflammatory responses, inducing endothelial dysfunction, and triggering mitochondrial oxidative stress. In contrast, BAT contributes to the maintenance of ovarian function by regulating thermogenesis, exerting anti-inflammatory effects, and increasing energy expenditure. Dysregulation of adipose tissue can lead to a decrease in antral follicle counts, anovulation, and dysregulated steroidogenesis, ultimately resulting in infertility and menstrual cycle irregularities. Current intervention strategies targeting adipose tissue dysfunction include BAT transplantation (primarily studied in animal models), exercise, dietary interventions, and pharmacotherapy, aiming to improve ovarian function and delay reproductive aging.

These mechanisms establish adipose tissue as a critical modulator of ovarian homeostasis, setting the stage for its pathophysiological roles in PCOS and reproductive aging (Fig. 3). Taken together, these findings collectively suggest a close relationship between obesity and ovarian function, with adipose tissue likely playing a key role. Adipose tissue may influence ovarian function.

Emerging evidence positions PCOS at the intersection of metabolic and endocrine dysregulation, with adipose tissue heterogeneity playing a pivotal role. Clinical investigations comparing age- and BMI-matched PCOS patients with controls have revealed distinct adiposity patterns: PCOS cohorts exhibit significantly elevated waist circumference (*Δ*+8.5 cm, *P *= 0.006) and reduced BAT activity compared to healthy individuals, as quantified by 18F-FDG PET-CT (SUVmax: 7.4 vs. 13.0 in controls, *P *= 0.047) [44]. Consistently, in an experimental PCOS rat model, BAT transplantation restored estrous cyclicity, reduced hyperandrogenism, and improved metabolic dysfunction, indicating a direct regulatory role of BAT in ovarian and systemic homeostasis [45]. This metabolic reprogramming manifests as a BAT-to-WAT shift, potentiating visceral adipose tissue (VAT) expansion. Experimental models have demonstrated that high-fat/high-sucrose (HFHS) feeding induces increased fat mass (2.12-fold) accompanied by adipocyte hypertrophy in VAT (23-fold area increase vs. controls) and PCOS-like ovarian dysfunction featuring hyperandrogenemia (+ 225% testosterone), disrupted estrous cyclicity, and aberrant follicular recruitment [42]. Notably, organelle dysfunction and inflammasome activation are more pronounced in visceral adipocytes than in subcutaneous adipocytes [46]. In other words, organ dysfunction caused by visceral fat tissue is more severe than that caused by subcutaneous fat tissue.

The aging process induces profound morphological and functional alterations in adipose depots, characterized by BAT “whitening” and visceral white adipose tissue (vWAT) dysfunction. BAT undergoes progressive lipid accumulation, transitioning from multilocular to unilocular lipid morphology, accompanied by downregulation of UCP1 expression and an increase in expression of preadipocyte markers [47]. This whitening process not only diminishes thermogenic capacity but also instigates chronic low-grade inflammation through necrotic adipocyte clearance and NLRP3 inflammasome activation [41, 48]. Concurrently, hypertrophic vWAT adipocytes activate organelle stress cascades involving endoplasmic reticulum dysfunction and mitochondrial reactive oxygen species (ROS) overproduction [46, 48]. These pathological changes culminate in systemic endocrine disruption, as evidenced by elevated levels of circulating proinflammatory cytokines, such as tumor necrosis factor-α (TNF-α) and IL-6, that permeate the ovarian microenvironment [49]. Clinical correlations have demonstrated parallel inflammatory escalation in the follicular fluid of obese women, suggesting that adipose-derived inflammatory mediators may accelerate ovarian reserve depletion [50].

Emerging interventions targeting adipose aging have demonstrated promising ovarian protective effects. BAT transplantation in aged murine models restores estrous cyclicity and ovarian follicular counts [51]. Moreover, through BAT transplantation, inflammation in epididymal white adipose tissue (eWAT) is reduced, which is supported by increased mRNA expression of anti-inflammatory genes (such as *Arg1, Chi3l3*, and *IL10*) and decreased expression of proinflammatory genes (such as *IL6, Mcp1*, and *Jnk1*) [52]. These results, although derived from epididymal fat in male models, highlight BAT’s potent anti-inflammatory capacity, which may similarly benefit the ovarian inflammatory microenvironment in females and support ovarian function. Notably, in long-lived animals, BAT activity increases, whereas in short-lived animals, BAT activity tends to decrease [53].

While these findings have established adipose–ovarian axis dysregulation as a hallmark of reproductive aging, critical knowledge gaps persist. The bidirectional relationship between chronic inflammation and BAT whitening remains enigmatic, particularly regarding temporal precedence and tissue-specific mediators. Furthermore, the relative contributions of endocrine (circulating adipokines) and paracrine (peripheral fat-ovary crosstalk) mechanisms warrant elucidation. Addressing these questions may unveil novel strategies for extending the reproductive lifespan through adipose tissue modulation.

Taken together, these findings indicate that the balance between BAT and WAT modulates ovarian function. Fortunately, relevant studies have shown that exercise, dietary interventions, and related pharmacological agents can promote the browning of WAT, leading to its conversion into BAT. This process can be induced by cold exposure or β3-adrenergic receptor stimulation, resulting in the production of UCP1-expressing brown-like adipocytes, also referred to as beige or brite adipocytes, which possess thermogenic capacity. Certain substances can improve fat balance by increasing the number of brown adipocytes, such as bone morphogenetic protein 7 (BMP7) [48]. Recent evidence further suggests that BMP7, derived from adipose-resident DPP4^+^ vascular cells, plays a key regulatory role in beige adipocyte aging and thermogenic decline, with potential systemic implications in metabolic and ovarian function [54].

## Gut–ovary crosstalk: microbial metabolites and immunoendocrine circuits in reproductive health

**Figure 4.**
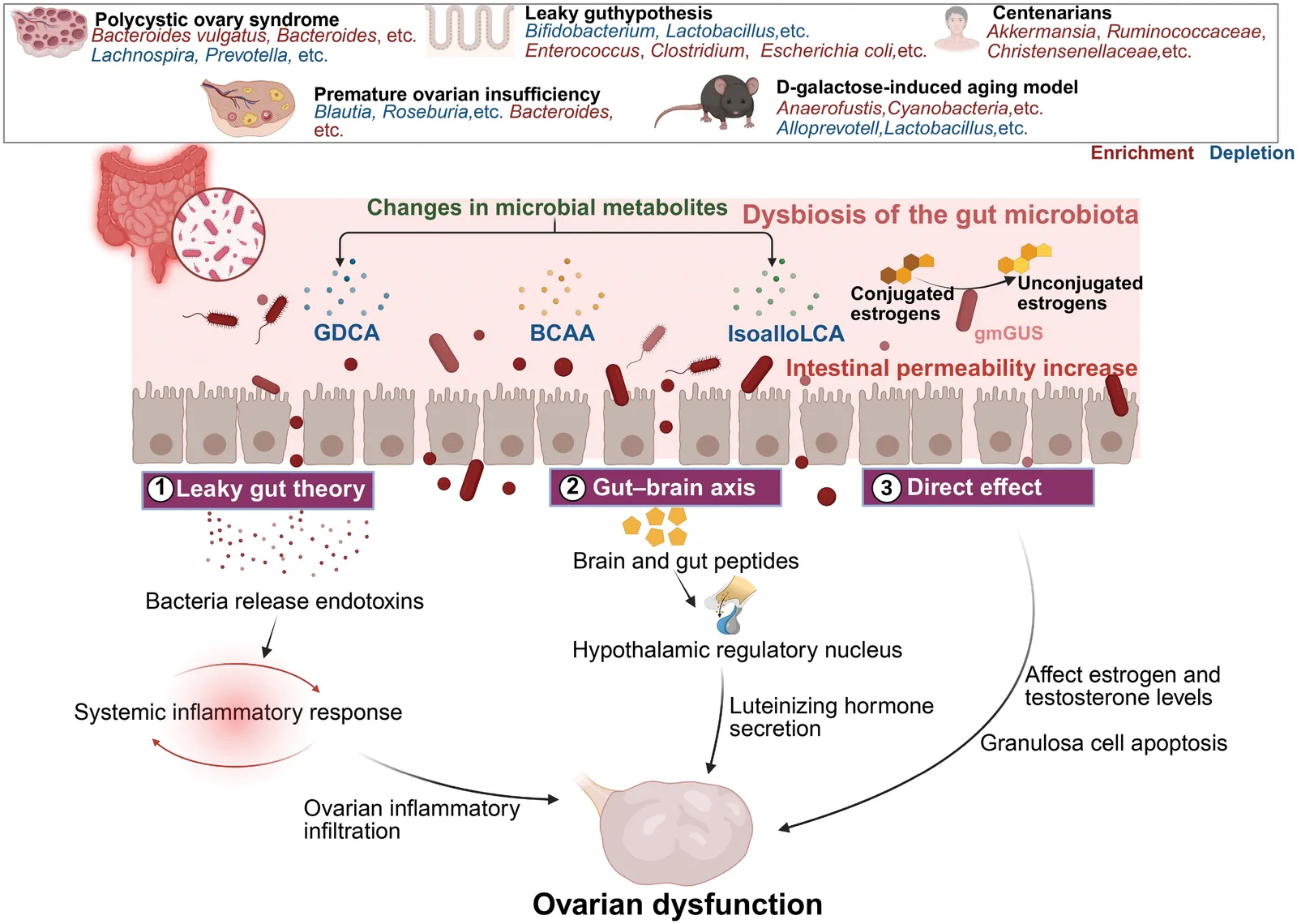
Mechanisms by which gut microbiota dysbiosis mediates ovarian dysfunction. Alterations in gut microbial composition have been implicated in various reproductive and aging-related conditions. Characteristic taxonomic patterns include enrichment of *Bacteroides vulgatus* and depletion of *Lachnospira* and *Prevotella* in PCOS, reduced abundance of SCFA-producing genera such as *Blautia* and *Roseburia* in POI, and increased *Akkermansia*, Ruminococcaceae, and Christensenellaceae in centenarians. In d-galactose-induced aging models, *Cyanobacteria* are enriched while *Lactobacillus* is depleted. These population- and model-specific microbial shifts may reflect differing stages or drivers of ovarian aging. Downstream of these microbial alterations, dysbiosis leads to functional disruptions via three interconnected mechanisms: (1) the “leaky gut” hypothesis, supported by the observed depletion of barrier-protective genera such as *Bifidobacterium* and *Lactobacillus*, and enrichment of endotoxin-producing taxa like *Enterococcus*, *Clostridium*, and *Escherichia coli*. Increased intestinal permeability allows microbial translocation, triggering systemic inflammation and ovarian immune infiltration. (2) The gut–brain axis, in which gut- or brain-derived peptides modulate hypothalamic regulation of luteinizing hormone secretion. (3) Direct endocrine effects, where microbial enzymes such as β-glucuronidase (gmGUS) regulate systemic estrogen levels by converting conjugated estrogens to active unconjugated forms, inducing granulosa cell apoptosis. Additionally, changes in key microbial metabolites—including glycodeoxycholic acid (GDCA), isoalloLCA, and BCAAs—mediate neuroendocrine and inflammatory pathways.

Emerging evidence has positioned the gut as a pivotal regulator of systemic homeostasis, with its bidirectional interactions extending to reproductive health [55]. In addition to its primary roles in nutrient metabolism and immune modulation, the gut performs endocrine-like functions through its microbial consortium [56]. The gut microbiota is considered an endocrine organ that is able to modulate the functions of various organs and their associated biological pathways [57]. This “microbial organ” predominantly features the phyla Bacteroidetes and Firmicutes, whose relative abundance crucially determines metabolic output [58]. Furthermore, emerging research suggests a close relationship between the gut and ovarian function (Fig. 4).

Clinical and preclinical studies have consistently demonstrated that gut dysbiosis is a hallmark of PCOS, although taxonomic alterations vary across experimental models. In patients meeting the Rotterdam criteria, PCOS is associated with diminished α diversity and distinct β diversity patterns, particularly those marked by overgrowth of *Bacteroides vulgatus*, which is a putative driver of microbial imbalance [59]. Mechanistically, fecal microbiota transplantation from PCOS donors or *B. vulgatus*-monocolonized mice recapitulates PCOS phenotypes in recipients, concomitant with reduced interleukin-22 (IL-22) and glycodeoxycholic acid (GDCA) levels. These inversely correlated biomarkers implicate bile acid-mediated modulation of intestinal group 3 innate lymphoid cells (ILC3s) as a gut-ovary signaling crosstalk [60].

In addition, a depletion of *Lachnospira* and *Prevotella*, and an enrichment of *Bacteroides*, *Parabacteroides*, *Lactobacillus*, *Fusobacterium*, and *Escherichia/Shigella* have been observed in PCOS patients [61].

The effect of the gut microbiota on PCOS development may involve three aspects. First, the “leaky gut” theory comes into play. Intestinal bacteria such as *Bifidobacterium* and *Lactobacillus* are often referred to as beneficial “probiotics” because they play an important role in preventing potentially harmful “bad bacteria,” such as *Enterococcus*, *Clostridium*, and *Escherichia coli*, from entering the systemic circulation through the colonic mucosal barrier [62]. Improvement in PCOS-like symptoms has been demonstrated via hormonal readiness, estrus cyclicity, and ovarian morphology after fecal microbiota transplantation (FMT) or *Lactobacillus* transplantation [56]. Second, the gut–brain axis plays a role. The gut–brain axis is a bidirectional communication system between the gut and the brain, and it is a neuroendocrine–immune network composed of the central nervous system, the enteric nervous system, the hypothalamic-–pituitary–adrenal (HPA) axis, and the gut. Brain–gut peptides, such as ghrelin, are involved in regulating the hypothalamic regulatory nucleus and luteinizing hormone secretion [63]. Finally, there is a direct effect. Gut microbiota can directly affect estrogen and testosterone levels [64]. This connection was first noted 40 years ago, and antibiotic supplementation has been shown to affect estrogen levels [65].

The gut microbiota establishes a dynamic endocrine dialog with host physiology through its enzymatic arsenal. Central to this interplay is microbial β-glucuronidase (gmGUS). The gut microbiota regulates estrogen levels by secreting gmGUS, an enzyme that converts conjugated estrogens into unconjugated forms within the gastrointestinal tract. This conversion facilitates the binding of estrogens to estrogen receptors, leading to subsequent signal transduction and downstream physiological effects [57].

Interestingly, hormone levels can also affect the gut microbiota. The composition of the symbiotic microbiota in male and female animals reportedly differs during puberty, indicating that sex hormone levels have a specific effect on the structure of the microbiota [56]. Such reciprocal regulation suggests a self-perpetuating cycle: gut dysbiosis may disrupt estrogen homeostasis, which in turn exacerbates microbial ecological imbalance—a phenomenon particularly relevant to PCOS pathophysiology characterized by concurrent hyperandrogenism and altered gut microbiota profiles.

Emerging evidence implicates gut microbial dynamics as key modulators of systemic aging processes. The gut microbiota has been reported to be involved in aging-related diseases and has become an important target for improving the health of elderly individuals [66]. Notably, studies in centenarians have revealed conserved microbial signatures enriched in *Akkermansia*, Ruminococcaceae, and Christensenellaceae abundance, suggesting that the presence of these taxa may contribute to longevity [67].

The ovary serves as a sentinel organ in female aging, and its functional decline orchestrates systemic senescence through complex microbiota crosstalk. Clinical profiling of POI patients has revealed striking gut microbial divergence, characterized by diminished abundance of short-chain fatty acid (SCFA)-producing *Blautia* and *Roseburia*, along with proliferating inflammatory taxa such as *Bacteroides*, patterns recapitulated in d-galactose-treated murine models showing *Cyanobacteria* expansion and *Lactobacillus* depletion [68, 69]. In addition, alterations in *Alloprevotella* and *Anaerofustis* abundances were associated with antral follicle dynamics, suggesting distinct microbial contributions to POI pathology [68].

Disrupted gut barrier integrity has emerged as a critical amplifier of ovarian aging. Microbial translocation triggers systemic inflammaging through endotoxin dissemination, a process potentiated by microbiota-dependent immune maturation deficits. Germ-free mice transplanted with human microbiota exhibit persistent intestinal immunodeficiencies, mirroring the leaky gut phenotype observed in POI patients [69, 70]. This breach permits infiltration of immunotoxic substances that disrupt ovarian steroidogenesis while activating follicular apoptosis pathways [71].

Metabolomic investigations have revealed microbial contributions to ovarian metabolic homeostasis. Centenarian-derived isoallolithocholic acid (isoalloLCA) has dual anti-inflammatory and antimicrobial properties, potentially counteracting age-related microbial shifts [72]. Conversely, POI patients exhibit branched-chain amino acid (BCAA) deficiency linked to impaired microbial biosynthesis, driving compensatory ketogenesis that exacerbates ovarian oxidative stress—a metabolic imbalance reversible through microbiota modulation [73, 74].

Therapeutic exploration has focused on microbiota–ovary crosstalk modulation. Established antiaging agents such as melatonin have pleiotropic effects, restoring microbial diversity while attenuating ovarian oxidative damage [75]. Emerging strategies target microbial metabolite pathways, with isoalloLCA analogs and BCAA-enriched probiotics showing preclinical promise. Nevertheless, critical gaps persist regarding sex-specific microbial trajectories and temporal windows for intervention, revealing a need for longitudinal studies to decipher dynamic host-microbe dialogs across ovarian aging stages.

At present, there are many ways to change the proportions and composition of the gut microbiota, including treatment with probiotics and caloric restriction. Therefore, we speculate that the gut microbiome plays a crucial role in aging and holds potential for therapeutic modulation. Improving the gut microbiota can help prevent and mitigate ovarian aging.

## Liver–ovary crosstalk: from androgen excess in PCOS to impaired hormone inactivation in ovarian aging

**Figure 5.**
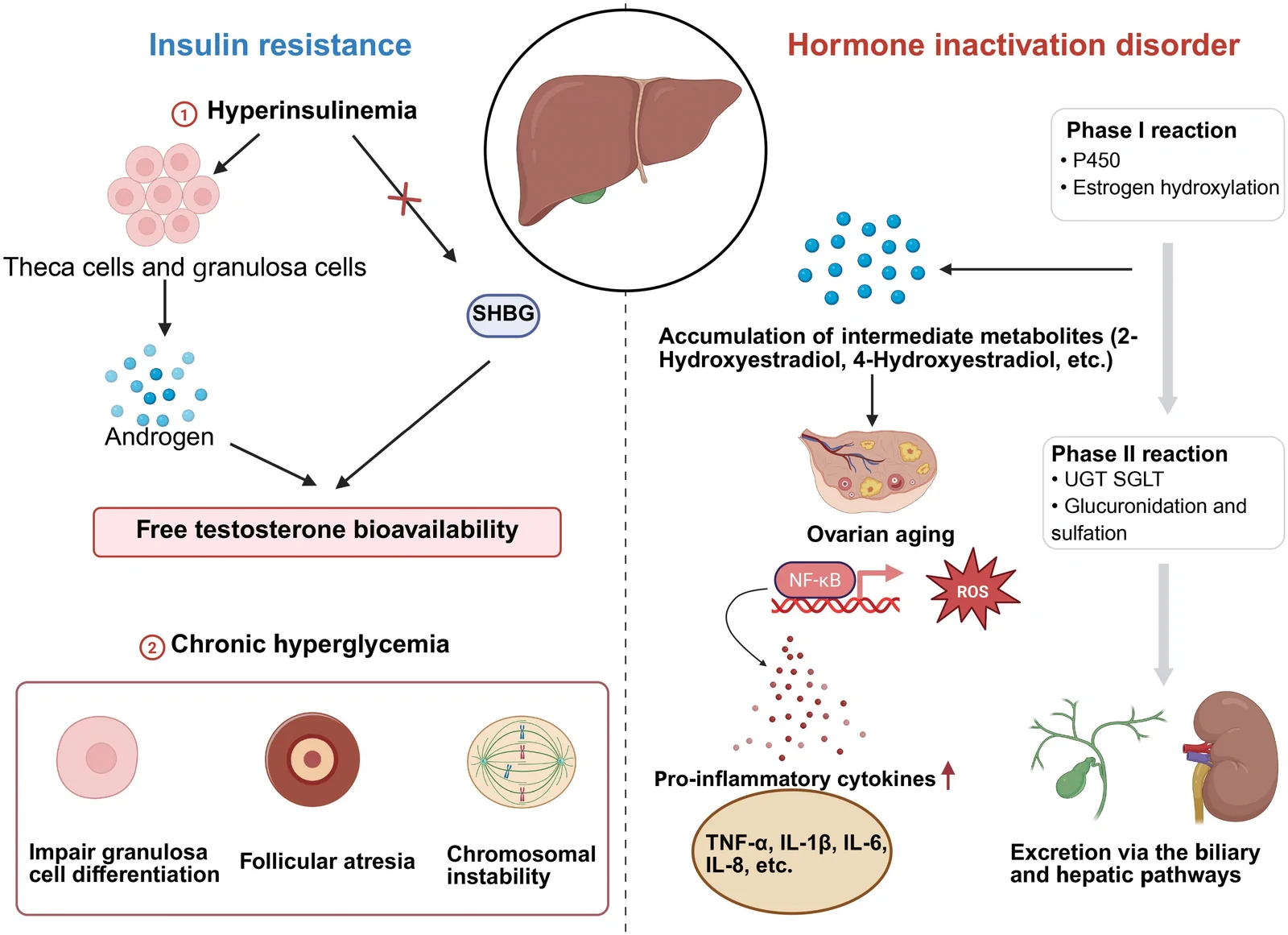
Mechanisms by which hepatic insulin resistance and hormone inactivation disorders contribute to ovarian dysfunction. Hyperinsulinemia promotes androgen production by theca and granulosa cells while inhibiting the synthesis of SHBG, leading to increased bioavailability of free testosterone and elevated ovarian androgen exposure. Meanwhile, chronic hyperglycemia resulting from insulin resistance further exacerbates ovarian dysfunction by impairing granulosa cell differentiation, promoting follicular atresia, and inducing chromosomal instability. In addition, hepatic hormone inactivation disorders also affect ovarian function. During the first phase (Phase I) of estrogen metabolism, hydroxylation is catalyzed by cytochrome P450 enzymes, followed by glucuronidation and sulfation reactions during the second phase (Phase II) to facilitate the biliary and hepatic excretion of metabolites and maintain hormonal homeostasis. When this metabolic process is impaired, intermediate metabolites such as 2-hydroxyestradiol and 4-hydroxyestradiol accumulate, accelerating ovarian aging. These metabolites activate the NF-κB signaling pathway in the ovary, increase the production of reactive oxygen species (ROS), and induce the release of pro-inflammatory cytokines (e.g. TNF-α, IL-1β, IL-6, IL-8), thereby creating a chronic inflammatory microenvironment that further damages ovarian function.

As a metabolic hub, the liver is indispensable for the regulation of glucose, lipid, and steroid hormone metabolism [76]. Interestingly, the liver plays a pivotal role in interorgan signaling pathways closely linked to ovarian function, which is essential for maintaining women’s reproductive health (Fig. 5). Investigating the role of the liver in metabolism and ovarian diseases provides valuable insight into disease mechanisms and offers potential therapeutic targets for managing these conditions.

Studies have shown that the prevalence of insulin resistance in PCOS patients ranges from 50% to 80%, which is significantly higher than that reported in the general population [2]. Insulin resistance is closely associated with metabolic disorders in individuals with PCOS, such as metabolic syndrome, type 2 diabetes, and cardiovascular diseases [76, 77]. Moreover, it significantly impairs reproductive function by disrupting follicular development and ovulation. During this pathological process, the liver, the central organ of insulin metabolism, plays a pivotal role in the metabolic disturbances and reproductive dysfunction observed in individuals with PCOS.

Emerging evidence highlights hepatic inflammation as a pivotal mediator of insulin resistance in individuals with PCOS. Experimental models have demonstrated marked activation of the canonical NF-κB pathway within hepatic tissues, driving excessive production of proinflammatory cytokines, including TNF-α, IL-1β, IL-6, IL-8, and MCP-1 [78]. These cytokines orchestrate systemic insulin resistance through dual mechanisms: direct inhibition of insulin receptor substrate (IRS) activity and suppression of Akt phosphorylation, which collectively impair the ability of insulin to suppress hepatic gluconeogenesis [79, 80]. Critically, emerging clinical observations reveal a paradoxical metabolic phenomenon in PCOS patients: as hepatic insulin resistance disrupts glucose homeostasis, ectopic lipid accumulation paradoxically progresses, driven by compensatory hyperinsulinemia [81]. Notably, hepatic lipid deposition leads to further deterioration of ovarian function. Nonalcoholic fatty liver disease (NAFLD), characterized by abnormal hepatic lipid accumulation, has been increasingly shown to have clinical associations with PCOS [82, 83]. Mechanistically, IL-1R1 activation amplifies this vicious cycle by promoting TNF-α/IL-6 overproduction and intensifying IRS/PI3K/Akt signaling inhibition, thereby reinforcing insulin resistance [84].

Clinical metabolomic profiling has identified dysregulated bile acid homeostasis as a characteristic feature of PCOS pathophysiology, with chenodeoxycholic acid (CDCA) demonstrating particularly elevated levels in affected individuals [85]. Notably, circulating CDCA concentrations exhibit a significant inverse correlation with insulin sensitivity indices [86]. Bile acids exert pleiotropic effects on glucose metabolism through the nuclear receptor FXR and the membrane receptor TGR5. Hepatic FXR activation suppresses gluconeogenic enzymes (G6P, PEPCK) while promoting glycogen synthesis, whereas TGR5 stimulates GLP-1 secretion via cAMP signaling to increase insulin sensitivity [87, 88]. However, PCOS-associated metabolic stress disrupts this hepatic regulatory pathway. Chronic hyperglycemia upregulates expression of CYP7A1, the rate-limiting enzyme in bile acid synthesis, establishing a self-perpetuating cycle of metabolic dysregulation [89]. Furthermore, bile acid imbalance induces oxidative stress through ROS overproduction in hepatocytes and Kupffer cells, impairing IRS/PI3K/Akt signaling and exacerbating insulin resistance [90].

Compensatory hyperinsulinemia arising from insulin resistance serves as a central driver of PCOS-related ovarian pathology. Insulin exerts direct gonadotropic effects on ovarian steroidogenesis by stimulating androgen production in theca and granulosa cells while suppressing hepatic hormone-binding globulin (SHBG) synthesis, thereby increasing free testosterone bioavailability [91, 92]. Concurrently, chronic hyperinsulinemia disrupts folliculogenesis through multiple mechanisms: impairing granulosa cell differentiation, promoting premature follicular atresia, and inducing chromosomal instability during oocyte maturation [93]. These endocrine perturbations establish a self-reinforcing cycle in which hyperandrogenemia exacerbates adipose tissue dysfunction and insulin resistance, in turn amplifying ovarian hyperandrogenism [91, 92, 94]. The bidirectional interplay between the metabolic and endocrine systems establishes a self-reinforcing pathogenic circuit, thereby sustaining the core reproductive and metabolic derangements characteristic of PCOS pathogenesis.

With aging, changes in hepatic metabolic capacity also impact ovarian function, particularly through a decline in the hormone inactivation process, leading to hormonal imbalances that further disrupt the ovarian microenvironment. Therefore, investigating the role of the liver in hormone inactivation is crucial for understanding the mechanisms underlying ovarian aging.

The liver maintains systemic sex hormone equilibrium through a tightly regulated two-phase enzymatic cascade. Phase I metabolism, which is primarily mediated by cytochrome P450 enzymes (CYP3A4 and CYP1B1), initiates estrogen inactivation via hydroxylation reactions [95]. This is followed by phase II conjugation through UDP-glucuronosyltransferases (UGTs) and sulfotransferases (SULTs), which increase metabolite hydrophilicity for efficient biliary and renal excretion [96, 97]. Notably, CYP3A4 exhibits significant sexual dimorphism, with baseline activity in females exceeding that in males by approximately 43%, an evolutionary adaptation potentially linked to cyclic hormonal demands [98]. Concurrently, CYP1B1 catalyzes the production of bioactive estrogen metabolites such as 2-hydroxyestradiol, which exert paracrine regulatory effects on the ovarian microenvironment [99]. Subsequent metabolic processing involves UGT-mediated glucuronidation and SULT2A1-driven sulfation, completing the estrogen clearance cascade [100, 101].

Age-related decline in liver function leads to impaired clearance of estrogen metabolites, particularly 4-hydroxyestradiol (4-OHE2), a reactive catechol estrogen generated via CYP450-mediated oxidation of estradiol (E2) [102]. This metabolic imbalance results in elevated systemic estrogen levels that potentially induce ovarian inflammatory responses through multiple mechanisms. Experimental studies have demonstrated that 4-OHE2 can generate ROS, causing DNA damage in pulmonary tissues [103], and similar oxidative stress mechanisms might operate in ovarian tissue. In the mammary epithelium, 4-OHE2 has been shown to activate the canonical NF-κB inflammatory pathway [104], suggesting a potential parallel pathway in ovarian cells. Although direct evidence of liver-derived metabolite accumulation in ovarian tissue remains unreported, we postulate that such metabolic crosstalk could induce ROS generation and inflammatory pathway activation. This hypothesis is supported by the observed proinflammatory transformation of the follicular microenvironment during reproductive aging. Notably, the NLRP3 inflammasome has been identified as a pivotal mediator of ovarian aging, and its activation is strongly correlated with functional decline [105]. The aging process is characterized by excessive secretion of proinflammatory cytokines, including IL-1β and TNF-α, establishing a chronic low-grade inflammatory state [20]. This immunosenescence-associated cytokine storm exacerbates tissue damage, disrupts follicular homeostasis, and ultimately leads to follicular depletion and ovarian insufficiency [106, 107], creating a vicious cycle that accelerates both ovarian and systemic aging.

The liver–ovary metabolic crosstalk has emerged as a critical regulator of reproductive aging in which hepatic dysfunction propagates ovarian senescence through estrogen metabolite-induced oxidative stress and NLRP3/NF-κB hyperactivation. Future investigations should prioritize hepatic interventions that prevent pathogenic accumulation of estrogenic intermediate metabolites, restoring metabolic equilibrium to block their inflammatory sequelae.

## Thyroid–ovary crosstalk: hormonal disruption, immune interference, and follicular dysfunction

Thyroid dysfunction disrupts female reproductive endocrine homeostasis through multiple pathways. Clinical studies have demonstrated that thyroid hormones (T3 and T4) directly regulate ovarian sex hormone synthesis and metabolism: patients with hyperthyroidism exhibit a two- to three-fold increase in total estrogen levels accompanied by markedly elevated sex SHBG levels, resulting in reduced free testosterone fractions, whereas hypothyroidism reduces SHBG levels, leading to decreased total estrogen and testosterone levels but elevated free hormone fractions. Concurrently, aberrant prolactin (PRL) secretion in patients with hypothyroidism suppresses gonadotropin axis function, contributing to ovulatory dysfunction [108]. These hormonal disturbances are closely linked to menstrual irregularities, with serum T4 levels in hyperthyroid patients positively correlated with the severity of menstrual disorders. Elevated T4/free T3 (FT3) levels are associated with ovulatory abnormalities and diminished follicular quality, whereas hypothyroidism is significantly linked to luteal phase deficiency and anovulation [108]. Notably, subclinical hypothyroidism (SH) impacts pubertal development: women with SH experience significantly earlier menarche (12.6 ± 1.2 years) than controls do (13.3 ± 0.8 years, *P* < 0.001), suggesting that thyroid function plays an early regulatory role in reproductive system maturation [109].

Thyroid hormones directly regulate ovarian follicular development through molecular mechanisms. Hypothyroidism reduces primordial follicle numbers by 60% and those of primary and preantral follicles by 40% in rats, impairing granulosa cell differentiation and fertility [110]. T3/T4 inhibit the expression of apoptosis markers (caspase-3, p53, and BAX) and activate PI3K/AKT signaling to increase granulosa cell survival [111]. Human ovarian cells express thyroid hormone receptors (TRs), and follicular fluid T3/T4 levels mirror serum concentrations, confirming direct thyroid–ovarian crosstalk [112, 113]. Clinically, follicular T4 levels correlate with oocyte yield (*r *= 0.352, *P* = 0.012), whereas T3 levels are associated with oocyte maturation rates, highlighting their critical roles in assisted reproduction [114].

Additionally, thyroid-related antibodies may interfere with the ovarian microenvironment by crossing the blood‒follicle barrier. A study revealed that levothyroxine (L-T4) treatment did not improve IVF outcomes in women with normal thyroid function who were positive for thyroid peroxidase antibody (TPOAb), confirming the association between thyroid autoimmunity and pregnancy outcomes [115]. These findings suggest that thyroid-related antibodies may affect ovarian function and reproductive outcomes through immune modulation and other nonhormonal pathways. Researchers have detected TPOAb and thyroglobulin antibodies (TGAb) in follicular fluid, which are strongly correlated with serum levels (*P* < 0.01) [112, 116]. Compared with negative controls, women with thyroid autoimmunity (TAI) exhibit significantly lower pregnancy rates [116]. Notably, ovarian tissue intrinsically expresses TSH receptors (TSHRs), suggesting possible cross-reactivity between thyroid antibodies and ovarian cells through molecular mimicry [117]. However, the precise pathogenic threshold requires further investigation.

Thyroid dysfunction and PCOS exhibit bidirectional interactions. Mendelian randomization analysis has demonstrated that PCOS increases the risk of hyperthyroidism (OR = 1.08, 95% CI = 1.02–1.13) [118], whereas Graves’ disease patients have a 47% greater incidence of PCOS (HR = 1.47) [119]. PCOS patients with Hashimoto’s thyroiditis (HT) display aggravated metabolic disturbances and exacerbated insulin resistance [120], which can be reversed by thyroxine supplementation or its analogs in PCOS model rats [121, 122]. Concurrently, thyroid autoimmunity correlates with diminished ovarian reserve: HT patients exhibit reduced antral follicle count (AFC) and anti-Müllerian hormone (AMH) levels (*P* < 0.05) [123], with 4%–30% of premature ovarian insufficiency cases showing thyroid antibody positivity versus < 10% in controls [124]. Mechanistically, T3 deficiency disrupts folliculogenesis by impairing thyroid receptor (TR)-mediated regulation of *CYP19A1* and mitochondrial genes (e.g. *PGC-1α*) [125], whereas anti-TPO antibodies accelerate ovarian reserve depletion [126, 127]. Notably, women aged 20–24 years with hypothyroidism face a 2.4-fold increased risk of diminished ovarian reserve [128], underscoring the critical role of thyroid homeostasis in reproductive health.

## Adrenal–ovary crosstalk: androgen metabolism, stress signaling, and ovulatory dysfunction

The adrenal gland and ovary form a dynamic regulatory network through hormonal interactions. Dehydroepiandrosterone (DHEA) and its sulfate (DHEAS), synthesized by the adrenal zona reticularis, serve as precursors for ovarian sex steroidogenesis via 3β-hydroxysteroid dehydrogenase (3β-HSD)-dependent conversion to active androgens and estrogens [129]. This adrenal‒ovarian synergy is particularly evident during perimenopause, during which declining DHEA levels correlates with accelerated ovarian reserve depletion, underscoring its role in follicular maintenance [129, 130]. However, pathological perturbations disrupt this equilibrium. Sleep deprivation-induced β2-adrenergic receptor (ADRB2) hyperactivity triggers premature primordial follicle activation through KITL–KIT/PI3K–mTOR signaling, culminating in ovarian reserve exhaustion [131]. Concurrently, chronic stress activates the HPA axis, increasing cortisol levels that upregulate gonadotropin-inhibitory hormone (GnIH) expression, thereby suppressing GnRH pulsatility and increasing anovulation risk by 70% [132–134]. Adrenal androgen dysregulation underlies diverse ovarian pathologies. In patients with adrenal insufficiency, hypoandrogenemia contributes to secondary ovarian insufficiency (SOI), with 38% of cases misdiagnosed as POI [135]. Androgen replacement therapy effectively restores the ovarian reserve, as evidenced by increased AMH levels [135]. Conversely, adrenal-derived hyperandrogenism drives PCOS pathogenesis, with 20%–30% of patients exhibiting elevated DHEAS levels [136]. Insulin resistance exacerbates this condition by increasing 17α-hydroxylase activity, perpetuating a vicious metabolic‒endocrine cycle [137]. Chronic inflammation and metabolic dysfunction further impair adrenal‒ovarian cross talk. TNF-α suppresses follicular survival through NF-κB activation, an effect that is partially reversed by dexamethasone (10 nM) treatment, which restores estradiol secretion [138].

## Cardiac–ovary crosstalk: vascular regulation, inflammatory pathways, and reproductive implications

Clinical observations have reinforced the idea of adrenal–ovarian interdependence. Thirty percent of congenital adrenal hyperplasia (CAH) patients develop PCOS, with ACTH levels > 100 pg/mL conferring a 3.2-fold increased risk, implicating hormonal dysregulation during disease progression [139]. Prenatal androgen exposure models have further demonstrated transgenerational PCOS transmission, where F1–F3 offspring exhibit characteristic reproductive and metabolic abnormalities, suggesting epigenetic reprogramming [140].

These findings mandate a paradigm shift toward dual-organ assessment in clinical practice. Routine evaluation of ovarian reserve (AMH, antral follicle count) in patients with adrenal disorders and adrenal hormone profiling (DHEAS, cortisol rhythm) in patients with ovarian pathologies could increase diagnostic accuracy. Unresolved questions persist regarding epigenetic modulation of granulosa cells by adrenal androgens and ovarian feedback on adrenal steroidogenesis. Addressing these knowledge gaps may yield novel biomarkers and dual-target therapies, ultimately advancing reproductive medicine from isolated organ management to systemic endocrine network modulation.

Growing bodies of evidence have suggested functional crosstalk between the heart and ovaries, although recent research has predominantly focused on unidirectional or indirect effects. Notably, reduced ovarian vascular density impairs folliculogenesis and fertility, whereas salidroside treatment enhances ovarian perfusion and rescues reproductive capacity in aged models [141], highlighting vascular homeostasis as a shared therapeutic target. In PCOS patients, chronic inflammation driven by splenic monocyte activation exacerbates postinfarction cardiac injury through macrophage infiltration and fibrosis [142]. Clinically, women with PCOS exhibit elevated risk of subclinical atherosclerosis due to hyperandrogenism, insulin resistance, and metabolic syndrome [143], underscoring opportunities for early cardiovascular intervention.

Complex congenital heart disease (CHD) patients display diminished ovarian reserve, as evidenced by reduced AMH levels [144], suggesting that chronic hypoxia indirectly compromises reproductive function. Delayed menarche in women with CHD implies that developmental vascular defects may interact with postnatal hemodynamic stress [145], although their relative contributions remain unquantified. While heart failure (HF)-associated inflammation leads to the release of circulating factors such as IL-6 and TNF-α [146], their distal effects on ovarian function have been poorly characterized. Current therapies, such as salidroside for ovarian revascularization [141], lack evaluation of cardiac cobenefits, reflecting a persistent organ-centric approach.

Critical knowledge gaps persist regarding direct interorgan signaling. Despite heart rate variability (HRV) reductions (e.g. SDNN, PNN50) indicating autonomic dysfunction in PCOS patients [147], no evidence has confirmed that neural pathways synchronize cardiac and ovarian impairment. Future studies must unravel multidimensional cardiac‒ovarian interactions through integrated neuroendocrine–immune analyses. Prioritizing the analysis of shared biomarkers could enable dual-organ intervention strategies, such as enhancing myocardial repair via ovarian angiogenesis or modulating follicular function via cardiac-derived signals. Additionally, systemic metabolic reprogramming via sex hormones and environmental stressors warrants exploration to establish lifespan-based preventive frameworks.

## Conclusion

The ovary operates as a convergence hub for multiorgan dialogs, through which distant physiological systems collectively calibrate reproductive competence from puberty to senescence. The hypothalamic command center emerges as the neural linchpin, and its GnRH pulsatility is distorted by age-related neuroinflammation and neurotransmitter imbalances. Chronic inflammation driven by adipose tissue signaling and alterations in adipokine secretion impairs ovarian function, whereas interventions promoting adipose remodeling have demonstrated potential in restoring ovarian homeostasis. Disruption of the gut microbiota is closely associated with PCOS and ovarian aging, and modulation of the gut microbiota may help attenuate ovarian aging. The liver influences ovarian function through metabolic disturbances, insulin resistance, lipid accumulation, and inflammatory responses, whereas age-related changes in hepatic metabolism accelerate ovarian aging.

The cardiac–ovarian axis reveals an unexpected phenomenon in which splenic monocyte-driven inflammatory cascades induce concurrent cardiac fibrosis, establishing a systemic inflammatory loop. Moreover, in the thyroid‒adrenal axis, hypothyroidism-induced SHBG level reduction synergizes with adrenal DHEA overproduction to exacerbate hyperandrogenic states, whereas HPA axis hyperactivity accelerates ovarian reserve depletion through cortisol-mediated GnIH signaling.

Three fundamental gaps persist in this evolving paradigm. First, a prevailing research paradigm is to dissect organ-specific mechanisms in isolation, neglecting the dynamic reciprocity within multiorgan circuits. Second, translational research is impeded by a lack of comprehensive human data linking organ-specific biomarkers to clinically relevant ovarian outcomes. Third, therapeutic development remains anchored in single-target approaches, overlooking the network pathophysiology of ovarian dysfunction.

Future investigations should prioritize three transformative strategies. Spatial multiomics mapping can be used to decode the hierarchical architecture of organ communication networks. Biomarker discovery may reveal combinatorial signatures, such as those integrating *Bacteroides* abundance, hypothalamic NF-κB activity, and serum CDCA levels, for the early prediction of ovarian aging. Most critically, the design of multiorgan therapeutics could pioneer a new era of precision interventions.

This systems-level reconceptualization positions ovarian health as an emergent property of interorgan synchrony. By targeting critical network nodes, we may ultimately decouple reproductive potential from chronological aging, offering revolutionary strategies against PCOS and age-related infertility through synchronized multisystem rejuvenation.
